# Cellular Stress Response Gene Expression During Upper and Lower Body High Intensity Exercises

**DOI:** 10.1371/journal.pone.0171247

**Published:** 2017-01-31

**Authors:** Andrzej Kochanowicz, Stanisław Sawczyn, Bartłomiej Niespodziński, Jan Mieszkowski, Kazimierz Kochanowicz, Małgorzata Żychowska

**Affiliations:** 1 Department of Gymnastics and Dance, Gdansk University of Physical Education and Sport, Gdańsk, Poland; 2 Department of Sport for all, Gdansk University of Physical Education and Sport, Gdańsk, Poland; 3 Department of Anatomy and Biomechanics, Institute of Physical Education, Kazimierz Wielki University, Bydgoszcz, Poland; 4 Department of Theory of Sport and Human Motorics, Gdansk University of Physical Education and Sport, Gdańsk, Poland; 5 Department of Life Sciences Gdansk University of Physical Education and Sport, Gdańsk, Poland; IRCCS E. Medea, ITALY

## Abstract

**Objectives:**

The aim was to compare the effect of upper and lower body high-intensity exercise on chosen genes expression in athletes and non-athletes.

**Method:**

Fourteen elite male artistic gymnasts (EAG) aged 20.6 ± 3.3 years and 14 physically active men (PAM) aged 19.9 ± 1.0 years performed lower and upper body 30 s Wingate Tests. Blood samples were collected before, 5 and 30 minutes after each effort to assess gene expression via PCR.

**Results:**

Significantly higher mechanical parameters after lower body exercise was observed in both groups, for relative power (8.7 ± 1.2 W/kg in gymnasts, 7.2 ± 1.2 W/kg in controls, p = 0.01) and mean power (6.7 ± 0.7 W/kg in gymnasts, 5.4 ± 0.8 W/kg in controls, p = 0.01). No differences in lower versus upper body gene expression were detected for all tested genes as well as between gymnasts and physical active man. For IL-6 m-RNA time-dependent effect was observed.

**Conclusions:**

Because of no significant differences in expression of genes associated with cellular stress response the similar adaptive effect to exercise may be obtained so by lower and upper body exercise.

## Introduction

High intensity exercise causes metabolic changes on many levels of human body altering the production of interleukins and heat shock protein [1–3], the availability of substrates, activation of metabolic enzymes [4], and others. All these changes start at the level of gene transcription. It is now understood that changes in genes expression caused by exercises occur primarily in genes associates with apoptosis and inflammation [1]. Considerable evidence demonstrates the influence of various types of exercise on inflammation [5, 6] and the expression of genes encoding heat shock protein [7, 8], thereby mediating the health benefits of episodic and prolonged exercise. The health promoting effects of exercise are associated with production of interleukins, elicited anti-inflammatory response trough inflammation [1], and increased stress tolerance.

It is now clear that measuring changes in the expression of genes associated with the cellular stress response, such as *HSPA1A* and *HSPB1*, synthesized by the cells of an organism in response to a variety of stimuli, including heat, oxidative, metabolic and chemical stress [9, 10]may be useful in determining physical workload [11] and intensity [12–14]or to monitor training [15]. The literature also suggests that type of physical work [16] and type of training [17] may affect the expression of genes encoding heat shock protein (HSP) and interleukins. In addition, athletes showed a decreased expression of *HSPA1A* mRNA as an adaptation to exercise [18]. According to Zeibig, et al. [19]and Maltseva, et al. [7], assessing the expression of genes encoding HSP may provide a valuable source of information about metabolic changes and the purposeful limitation of applied training loads. Such measurements may be particularly important for athletes, especially high-level athletes, among whom the variability of physiological indicators is not very dynamic. It is also possible that such changes are associated with specific types of training that accompany various sporting disciplines.

Determining of pro- and anti-inflammatory cytokines after exercise is important in terms of health [5] and physical effort. Optimizing the inflammatory and immune responses may help to promote health and prevent overtraining [5]. Ziemann, et al. [20]suggested that decreasing inflammation and increasing anti-inflammatory factors is very important for adaptation to exercise. Moreover, high intensity interval exercise can induce a greater increase in IL-6 protein compared with continuous moderate-intensity exercise [21]. Reports in the literature indicate that production of pro- and anti-inflammatory proteins accompanied the stress response, regardless of the kind of stressors (e.g., temperature, physical effort) or signalling pathway activation, including the *HSF-1* and *NF-kB* pathways [2, 3]. Changes in the expression of these genes is the result of changes in signalling via these pathways.

Only one study in the literature has associated changes in protein levels of IL6 and IL10 after intense judo exercises involving the lower and upper body separately [22]. According to these authors it is difficult to investigate lower and upper body high intensity performance and immunometabolic responses because there is a weaker adaptation to exercises in athletes that focus on the upper body [22] as well as in sedentary people. The authors suggest that despite the higher performance in lower body, the inflammatory response did not differ between exercise performed using the upper body. Importantly, this research is based entirely on the results obtained by observation of protein levels. There are no data in the literature reporting changes in the expression of genes encoding interleukins and heat shock proteins during intense exercise performed by the lower and upper body.

Artistic gymnasts must demonstrate skills in accordance with accepted techniques by performing exercises on various apparatuses, forcing athletes to demonstrate extremely high levels of explosive power and endurance-strength capabilities without significantly mobilizing the aerobic processes [23]. The duration of gymnastics exercises at the highest level in all competitive apparatuses, except vaulting, is small (40 to 70 s). In gymnastics, the anaerobic metabolism is considered the main energy supplier. This maximal, short duration effort categorizes gymnastics as a high-intensity, anaerobic sport in which the upper and lower body should be well-trained [24–26]. Moreover, our research involving athletes and non-athletes suggests that the response to exercise may be different. Furthermore genes tested in this study are easy inducible by similar stimuli, such as oxidative stress, muscle damages but also are sensitive to transcriptional factors (*HSF-1* mainly induce heat shock protein and *NF-kB* especially *IL6*). It is possible that changes in temperature, oxidative stress, muscle damages could be similar after lower and upper body Wingate test, which is performed with maximal intensity but in short time.

In many sports anaerobic possibilities within upper body are more important than within lower body [27]. Unfortunately there are not much data in the literature associated with response to exercises performed by upper body, especially in response to exercise at molecular level. To our knowledge it is the first study in which influence of upper and lower body exercises on stress-related genes expression in athletes and non-athletes was compared.

Thus, the aim of this study was to evaluate the changes between lower and upper body high intensity exercise in expression *HSPA1A*, *HSPB1*, and *HSF-1* as well as *NF-kB*, *IL6* and *IL10* in athletes and non-athletes. Based on the available literature obtained for protein level we hypothesize that regardless expected high differences between upper and lower body Wingate Test changes in gene expression may be little in leukocytes in both groups and that these changes may be smaller in athletes because of their good adaptation to high intensity exercise.

## Methods

### Ethics statement

This study was approved by the Bioethics Committee for Clinical Research at the Regional Medical Chamber in Gdańsk and conducted according to the Declaration of Helsinki. All participants gave their written consent to participate in the study and were informed about the purpose and test procedures as well as about the possibility of withdrawal of consent at any time for any reason.

### Participants

A group of 14 elite male artistic gymnasts (EAG) aged 20.6 ± 1.9 years and 14 physically active men (PAM) aged 20.2 ± 1.2 years participated in this study. The gymnasts trained for 2 sessions per day 6 days per week (25–28 hours). Physically active men declared regular recreational participation in sports such as running, swimming, judo, and team sports (on average, 2–3 times a week for a duration of 45 minutes). This group, untrained in professional gymnastics, served as a control group with respect to long-term gymnastic training.

The participants had a normal health status during the previous three months (no injuries to the bone or the muscle tissue; no intake of drugs during the study; negative medical history regarding disorders of the cardiovascular system, autonomic nervous system, mental disorders, craniocerebral traumas, and other diseases that might directly affect the obtained results). The participants were informed of the nature and possible inconveniences associated with the experiment. Descriptive physical characteristics are shown in Table 1 (individual data can be found in Table A in S1 File).

**Table 1:** Physical characteristics of the elite artistic gymnasts (EAG) and physically active men (PAM) in mean and standard deviation (SD).

	EAG	vs	PAM
**Body mass (kg)**	68.3 ± 6.5	*	73.7 ± 6.9
**Height (cm)**	171.4 ± 4.1	*	177.6 ± 4.1
**Body mass index (kg/m^2^)**	23.3 ± 1.5		23.4 ± 1.9

* *p* < 0.05, difference between gymnast (EAG) and control (PAM).

Comparisons of subjects’ anthropometric characteristics did not show significant differences in body mass index (BMI) (p = 0.75, *F*_1,43_ = 0.13). However, the PAM had a higher total body mass (p<0.05, *F*_1,43_ = 5.2) and height (p< 0.001, *F*_1,43_ = 21.2) than the EAG.

### Experimental overview

The study consisted of two parts. First was the measure of anaerobic components of fitness using Wingate Anaerobic Tests (WAnTs) and the second was the assessment of gene expression in blood samples collected during the first part. Each part of the study is described in additional detail below. Prior to any testing, all participants attended 1 hour familiarization session, one week prior to experiment to ensure that all participants were familiar with the testing equipment and procedures. All participants performed lower and upper body Wingate Test. Actual measurements began with lower body WAnT. Before the test, venous blood was taken at rest as well as 5 and 30 minutes after completion. Because of regular training in gymnasts from Monday to Saturday our sportsmen performed lower and upper body exercises on Mondays after 48 hours of brake. One week later, participants completed the upper limb WAnT and blood samples were collected at rest before and up to 5 and 30 minutes after completion of the test [7]. For 48 hours prior to testing, participants were asked to refrain from exhaustive exercise, to maintain their normal dietary habits, and to come to the laboratory in a euhydrated state. The participants were instructed to avoid caffeine, alcohol and soda one month before experiment.

#### Measurement of anaerobic components of fitness: lower body and upper body wingate tests

The lower body WAnT was conducted on a cycle ergometer (Monark 894E, Peak Bike from Sweden). For each participant, the saddle height was adjusted so that the knee remained slightly flexed after the completion of the downward stroke (with final knee angle approximately 170–175°). Toe clips were used to ensure that the participants’ feet were held firmly in place and in contact with the pedals. Before any experimental testing, each individual completed a standardised warm-up on the cycle ergometer (5 min at 60 rpm, 1W/kg). Each participant was required to pedal with maximum effort for a period of 30 s against a fixed resistive load of 75 g/kg of total body mass as recommended by [28].

The upper body WAnT was conducted on a hand cycle ergometer (Monark 891E). Participants sat in a chair fixed to the ground and were advised to keep their feet flat on the ground and remain seated throughout the WAnT. The seat height and back rest were adjusted so that with the crank position on the opposite side to the body and the hand grasping the handles, the elbow joint was almost in full extension (140–155°) and the shoulders in line with the centre of the ergometer’s shaft. A standard resistive load equivalent to 50 g/kg of total body mass was applied for each participant [29]. Before the test, the participants completed a warm-up that involved 5 min of arm cranking using a power output of 1 W/kg and a crank rate of 60 rev/min.

For both lower body WAnT and upper body WAnT, each participant was instructed to cycle as fast as possible and was given a 3 s countdown before the set resistance was applied. Verbal encouragement was given to all participants to maintain their highest possible cadence throughout both WAnTs. Both cycle ergometers were connected to a PC to allow data capture via the MCE 5.1 software. The following WAnT variables were measured: peak power (W) and relative peak power (W/kg) was calculated as the highest single point of power output (recorded at 0.2 s intervals); mean power (W) and relative mean power (W/kg) was the average power output during the 30 s test.

#### Sample collection and genetic research methodology

Blood for genetic research was collected three times on the day of each test (immediately before and up to 5 and 30 minutes after Wingate Test completion). To eliminate erythrocytes, 2 ml of venous blood was treated with red blood cell lysis (RBCL) buffer (A&A Biotechnology, Gdynia, Poland).The remaining white blood cells were lysed using Fenozol (A & A Biotechnology, Gdynia, Poland). Further isolation of RNA was carried out by a chemical method as described by Chomczynski and Sacchi [30]. Purity and concentration of the isolated RNA was determined by spectrophotometry (Eppendorf BioPhotometer Plus, Germany). cDNA synthesis from 2 μg RNA was performed using the TranscriptMe Kit, containing oligo dT and random hexamers (Blirt, Gdańsk, Poland).

For the analysis of genes expression, real-time PCR (LightCycler 480 II, Roche, Poland) was applied twice in three replicates for each sample using a LightCycler polimerase (Roche, Poland). The temperature-time profile of the reaction was consistent with the manufacturer's instructions. For each reaction, a melt curve analysis was performed. The TATA box protein (*TBP*) was used as a housekeeping gene. To amplify the genes, the following primer sequences were applied (Table 2).

**Table 2:** Primers used in PCR reactions.

	Primers
*TBP*	R: TTCGGAGAGTTCTGGGATTGTA
	F: TGGACTGTTCTTCACTCTTGGC
*HSPA1A*	R: TCTGTCGGCTCCGCTCTGAGAT
	F: ACTCCCGTTGTCCCAAGGCTTC
*HSPB1*	R: GAGGAAACTTGGGTGGGGTCCA
	F: AAGGATGGCGTGGTGGAGATCA
*HSF-1*	R: CAGGAGCTTGGAGTCCATGCA
	F: GAGCAGCTCCTTGAGAACATC
*IL6*	R: GACATCAAGGCGCATGTGAAC
	F: TCCACGGCCTTGCTCTTGTTT
*IL10*	R: AATTCGGTACATCCTCGACGG
	F: GAATCCAGATTGGAAGCATCC

F, forward primer; R, reverse primer.

### Statistical analysis

Descriptive statistics included mean ± SD for all measured variables. The normality of distribution was checked with the Shapiro-Wilk’s test. Gene expression data were collected and relative gene expressions were analysed in Excel 2010. In order to calculate the level of gene expression, the method of Schmittgen and Livak [31] was used, and data were then transformed from 2^ to linear value. To assess statistical significance of the changes in genes expression before and after exercise (WAnT), a repeated measures analysis of variance (ANOVA) was used. To determine the differences in genes expression after the lower and upper body WAnT, a paired t-test was calculated. To calculate differences between groups, two-way ANOVA of repeated measures (2 groups x 3 measures) was applied. Post-hoc analyses were implemented when appropriate with Tukey’s post hoc test. The data was presented on the Figures as 2^fold changes (2^FC). A one-way analysis of variance (ANOVA) was used to determine the difference between PAM and EAG in performance characteristics in all variables during the WAnTs. In addition, the effect size of the researched relations was estimated (Cohen’s d values). All calculations and graphics were performed using GraphPad Prism 6.0 (ftx.pl/program/graphpad-prism). Differences were considered statistically significant differences at a level of p≤0.05.

## Results

Characteristics of lower and upper body Wingate Anaerobic Test (WAnT) assessments for all participants are summarized in Table 3 (individual data can be found in Table B in S1 File).

**Table 3:** Lower and upper body Wingate Anaerobic Test (WAnT) characteristics of elite artistic gymnasts (EAG) and physically active men (PAM) in mean and standard deviation (SD).

WAnT	EAG		PAM		Cohen’s *d*
	Mean ± SD	(95% CI)	Mean ± SD	(95% CI)	*EAG / PAM*
***Lower body***					
Absolute peak power (W)	753.6 ± 114.9	(690.4–816.8)	777.1 ± 127.2	(702.6–851.5)	0.19
Absolute mean power (W)	582.5 ± 75.5	(539.2–626.4)	609.9 ± 97.2	(553.7–665.9)	0.31
Relative peak power (W/kg)	11.1 ± 1.0	(10.6–11.6)	10.5 ± 1.4	(10.2–11.3)	0.49
Relative mean power (W/kg)	8.6 ± 0.6	(8.2–8.9)	8.3 ± 1.0	(7.6–8.9)	0.36
***Upper body***					
Absolute peak power (W)	614.0 ± 90.3	(562.2–666.5)	542.2 ± 126.8	(473.2–611.1)	0.65
Absolute mean power (W)	475.5 ± 69.2*	(435.5–515.5)	419.2 ± 70.9	(373.4–460.6)	0.80
Relative peak power (W/kg)	9.1 ± 1.4 *	(8.5–9.5)	7.3 ± 1.3	(6.6–8.1)	1.33
Relative mean power (W/kg)	7.0 ± 0.6 *	(6.6–7.3)	5.6 ± 0.8	(5.2–6.1)	1.97

WAnT, Wingate anaerobic test.

* *p* < 0.001, difference between EAG and PAM.

In both groups there were significantly higher values after lower body exercise. Absolute peak power differences between means ranged from 139.6 W in elite male artistic gymnasts (EAG) and 234.9 W in physically active men (PAM) (p < 0.01). The range for absolute mean power was from 107 W in EAG to 190.7 W in PAM (p < 0.01), 1.9 W/kg to 3.2 W/kg (p < 0.01) for relative peak power, and 1.6 W/kg and 2.7 W/kg (p < 0.01) for relative mean power. Differences between all analysed parameters were higher for the PAM group.

Though not significant, PAM produced higher absolute peak power (p = 0.60, *F*_1,26_ = 0.26) and mean power (p = 0.41, *F*_1,26_ = 0.67) during the lower body WAnT compared to their gymnast counterparts. Relative peak power (p = 0.25, *F*_1,26_ = 1.37) and mean power (p = 0.33, *F*_1,26_ = 0.77) were similar in both groups. In contrast, the gymnasts produced significantly higher absolute mean power (p<0.042, *F*_1,26_ = 4.55) relative peak power (p < 0.001, *F*_1,26_ = 16.16) and mean power (p < 0.001, *F*_1,26_ = 25.72) during the upper body WAnT compared to the PAM group. Absolute peak power (p = 0.08, *F*_1,26_ = 3.25) of the upper body was also higher in the gymnasts, but the difference was not significant.

### Gene expression

**Fig 1:**
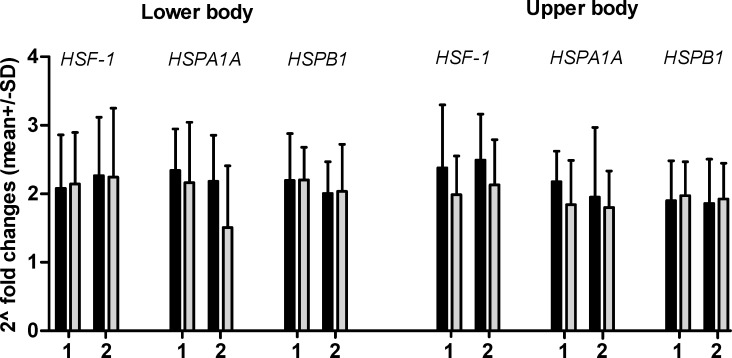
2^FC in genes expression after lower and upper body exercise in EAG (gray bars) and PAM (dark bars). 2^FC between 5 min after and rest value.2^FC between 30 min after and rest value.

**Fig 2:**
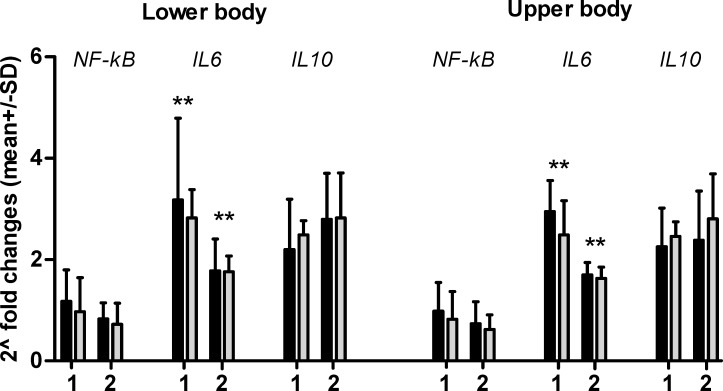
2^FC in genes expression after lower and upper body exercise in EAG (gray bars) and PAM (dark bars). X-fold changes were calculated as B/A 2^FC between 5 min after and rest value.2^FC between 30 min after and rest value. *significant differences between 5 and 30 min post body exercise.

The changes in expression of *HSF1*, *HSPB1*, and *HSPA1A* m-RNA after supramaximal lower and upper body effort in both groups are presented in Fig 1 and *NF-kB*, *IL6*, *IL10* m-RNA in Fig 2. Changes in expression are presented as 2^-fold changes (2^FC) (Figs 1 and 2, Tables C and D in S1 File).

Applied supramaximal effort in the lower and upper body caused up-regulation of all genes in both groups. Changes in expression *HSF-1*, *HSPA1A* and *HSPB1* m-RNA was similar after lower and upper body exercises in EAG and PAM. There were no significant differences between this groups but the expression of *HSF-1* and *HSPA1A* m-RNA was slightly lower in EAG after exercise performed by upper body compare to PAM.

Changes in *IL6*, and *IL10* m-RNA 5 and 30 min post supramaximal exercise lower and upper body are presented in Fig 2.

After supramaximal effort in the lower and upper body the changes in *IL6*, *IL10* m-RNA were different. Generally, there is a noted tendency toward lower expression of *IL6* and higher expression of *IL10* m-RNA 30 min after exercise. It can be seen by downregulation of expression of *IL6* through up-regulation *IL10* m-RNA after lower and upper body exercise. The decrease in *IL6* m-RNA 30 min after exercises compare to 5 min was significant in both groups and after lower and upper body exercises amounted to: 2^2.80-fold to 2^1.77-fold p = 0.001 and 2^2.49 to 2^1.43 p = 0.02 in EAG and 2^3.17-fold to 2^1.8 p = 0.05 and 2^2.95 to 2^1.7-fold p = 0.015 in PAM.

Differences between lower and upper body exercises for *NF-kB*, *IL6* and *IL10* m-RNA were not significant in the EAG or PAM groups 5 or 30 min after exercise (ANOVA 2-way). There were also no significant differences between EAG and PAM.

## Discussion

To our knowledge this is the first study in which lower and upper body high intensity exercise is analyzed on the transcript level. We hypothesized that WAnT results would be higher after lower body exercise for all indicators. This hypothesis was correct, all indicators were on higher after lower body exercise (Cohen’s d strong for all measures and groups, Table 3). Compared to physical active men untrained in gymnastics, the athletes had significantly greater relative peak and mean power in the upper body during WAnT assessment, indicating specific adaptation of anaerobic capabilities. People in control group were significantly taller and heavier than our gymnast, but BMI was the same in both groups, which reduced the differences during WAnTs. Relatively fewer exercises engage the lower body as they are mainly utilized in short, explosive efforts. This may help to explain the lack of differences in relative peak and mean power between EAG and PAM observed in the lower limb WAnT. However, it is difficult to unequivocally state that the results observed in the EAG studied here are typical for this group of athletes. For example, in a study of anaerobic capabilities in elite French gymnasts, [32] showed that relative peak (13.4 ± 1.3 W/kg) and mean power (9.6 ± 1.0 W/kg) were higher in the lower body than in the upper body. Similar higher relative peak results (11.7 ± 1.2 W/kg) were also observed in elite Greek gymnasts, although this difference was less pronounced in comparison to the difference presented here [23]. In contrast, anaerobic capabilities of the upper body in EAG appeared similar to those described in the previously mentioned international French gymnasts [32] (relative peak power: 9.2 ± 1.1 W/kg; relative mean power: 6.6 ± 0.6 W/kg) as well as to athletes training in judo [33] and wrestling [34].

Our results associated with heat shock protein and interleukin genes expression showed no significant differences between lower and upper body exercises. However, slightly higher differences between groups was observed after exercise performed by upper body (mainly in *HSF-1* m-RNA). This may indicate specific adaptation gymnasts to exercises performed by upper body. The differences in genes encoding IL6 and IL10 was also no significant both between lower/upper exercises and between groups. Similar data associated with inflammation were reported by Lira, et al. [22], obtained for interleukin on seven male judo athletes who performer repeatable Wingate testing on lower an upper body exercise. The authors suggest no differences in plasma interleukin 6 and 10 after lower and upper body exercise. Independent that our study was performed on transcript level in leukocytes the observed changes are similar and up-regulation in genes expression can suggest, that these cells participate in total inflammation. The response to physical exercise is not only local but systemic, making analysis of relative gene expression in peripheral blood leukocytes a reasonable indicator of the whole body response to our experimental intervention [7].

Changes in the expression of genes such as *HSPA1A* and *HSPB1* are hard to interpret. On the one hand, their overexpression suggests the level of stress load [35], whereas a decrease in their expression after exercise might be associated with the absence of adequate protection against factors disrupting metabolic functions within the body. In addition there are only few reports referring to changes of the expression these genes after acute exercise [1] and between athletes and active people.

The differences between EAG and PAM in gene expression examined in our study were also no significant, but several directions can be distinguished. The lower activity in EAG was for *HSF-1*, *NF-kB*, *HSPA1A*, *IL6* m-RNA. These differences are consistent with the results obtained by several authors, which reports significantly lower expression of genes associated with heat shock protein especially in *HSPA1A* m-RNA in athletes compare to sedentary people [11, 36]. Our study showed lower expression of *HSPA1A* in athletes, especially after upper body exercise and are consistent with results reported by Buttner, et al. [11] and Neubauer, et al. [36], who reported a lower level of expression of these genes in athletes in compare to untrained people. It can be postulated, that for athletes stress load caused by lower and upper Wingate Anaerobic Test was smaller [19]. Moreover, lower gene expression results after upper body exercise in athletes accompanied with better results of Wingate test. We suspect that the tested gymnasts may have high adaptation of the upper limbs to exercise as effect of specific training. On the second hand control group had good physical possibilities, and they should’t be treated as sedentary people. It is possible that there are differences in activation of endocrine system (especially stress hormones) between groups. In the literature there are interesting studies in which cortisol level after four WAnTs was investigated. Moreover, changes in cortisol level occurred only in people regularly trained but not in control group [37, 38]. For this reason cortisol level in control group was not analyzed during high-intensity intermittent training [39]. Since it is known that cortisol level may influence IL1 and IL6 values, these differences between groups may also affect genes expression [40].

Two of the examined transcriptional factors are associated with the stress cellular response [3]. *HSF-1* is responsible for activation of *HSPA1A* and *HSPB1*, among other things, while *NF-kB* enhances the inflammatory response, including *IL6* activation and is known as redox-sensitive transcription factor [41]. There are some reports in the literature that associate downregulation of *NF-kB* expression with *IL10* m-RNA [42–44]. In our study, this relationship was also slightly, but higher expression of *IL10* 30 min after exercises caused in slightly lower expression *NF-kB* m-RNA. Additionally, this effect was similar after lower and upper body exercise and caused in significantly lower expression *IL6* m-RNA. Decrease of *IL6* m-RNA after exercise was also reported in the literature. Nieman, et al. [45] investigated time effect of *IL10* and *IL6* expression after WAnT and reported 2.7-fold increase for *IL10* and 0.8-folddecrease of *IL-6* m-RNA in the same time (from immediately after to 1 hour after). Two hour after exercise there were no changes in *IL6* m-RNA after acute exercise. In our study significant effect time for *IL6* m-RNA was observed after lower and upper body exercise in both groups.

The changes in gene expression results following upper Wingate Anaerobic Test in PAM and EAG presented in this study suggest no time dependent changes in expression of *HSF1* during half hour after exercise. It is well-known that *HSF-1* is an activation domain of genes encoding *HSP*. Because of the fact that slightly higher mean changes in *HSF-1* were observed 30 min after exercise, it is possible, that changes in *HSPA1A* and other genes may be higher later. This suggestion is related to methodology as magnitude of changes may be dependent on the time at which the determination is made.

Moreover, the results are not consistent with the results published by Radom-Aizik, et al. [6]. The authors reported that 30-min of acute exercise affected *HSP* genes expression only in untrained men. This differences may be associated with the short time frame and aerobic characteristic of the applied effort and/or relative lower load for studied athletes as well as better adaptation to acute exercise than in the gymnasts examined here.

Data in the literature indicate a higher stability of *HSPB1* m-RNA during and after various exercises. Maltseva, et al. [7] reported no changes in expression *HSPB1* after 30 min of moderate intensity exercise. This study on basketball players indicated that after plyometric training, overexpression of *HSPB1* occurs later and not immediately post effort [8]. In present study up-regulation of *HSPB1* m-RNA appears 5 min after exercises and level of m-RNA was similar after 30 min after exercise. The study on plasma level HSP70 and HSP27 showed, that there are strong correlation between HSP70 and HSP27 after exercises performed with different intensity [46]. Cited authors concluded, that changes in these proteins are associated with intensity and duration of exercise. In our study *HSPB1* m-RNA after upper body exercises was similar to obtained after lower body and was not downregulated in athletes, as *HSPA1A*. It is possible that *HSPB1* plays a very important role in the degradation of damaged proteins during physical effort [16, 47] and that the demands of small HSP synthesis occur longer. Furthermore, transcriptional activity can be stimulated by muscle damage [48]. Therefore, greater muscle damage can occur because of higher possibilities to exercise (significantly higher relative and mean power in gymnast after upper body exercise and slightly higher expression in *HSPB1* m-RNA.

In conclusion, independently from the higher performance in lower body exercise, there was no significant difference in inflammatory gene expression (*IL6*, *IL10*) and genes encoding heat shock proteins (*HSPA1A*, *HSPB1*) between lower and upper body exercise and between groups. Therefore, stress load after lower and upper body exercises was similar and health benefits as well as adaptive changes to exercise can be obtained by prolonged exercise performed both for lower and upper body. In the literature there is information, that HIIT exercise can be beneficial in areas of the body other than those directly involved in the exercise [49]. Our results indicate, that this sentence is also associated with adaptation to training on molecular level.

### Practical application

Based on obtained results it can be suggested that for the maintenance of adaptation on molecular level, lower and upper body training could be useful. It is useful information for sport practice, that during forced breaks in the exercises performed by upper limbs appropriate continuation of training by lower limbs would be recommended, and vice versa.

## Supporting Information

S1 File Relevant data underlying the findings described in manuscript. Table A.xlsx. Individual physical characteristic of PAM and EAG.Table B.xlsx. The raw data and basic statistics of lower and upper body WAnTs.Table C.xlsx. Individual results and basic statistics of genes expression (PAM and EAG).Table D.*.PZF. Statistics and results used for analysis of genes expression.
(ZIP)

## References

[1] Gjevestad GO, Holven KB, Ulven SM. Effects of Exercise on Gene Expression of Inflammatory Markers in Human Peripheral Blood Cells: A Systematic Review. Current cardiovascular risk reports. 2015;9(7):34 Epub 2015/05/26. 10.1007/s12170-015-0463-4 26005511 PMC4439514

[2] Rutkowski R, Pancewicz SA, Skrzydlewska E, Hermanowska-Szpakowicz T. Biology of nuclear transcription factor NF-κB. Alergia Astma Immunologia. 2005;10:125–31.

[3] Szołtysek K, Janus P, Widłak P. The NF-kB dependent cellular signaling pathway and its interference with p53 and HSF1-depentent pathways. Advances in Cell Biology. 2011;38:159–75.

[4] Buchheit M, Laursen PB. High-intensity interval training, solutions to the programming puzzle. Part II: anaerobic energy, neuromuscular load and practical applications. Sports Med. 2013;43(10):927–54. 10.1007/s40279-013-0066-5 23832851

[5] Kimsa MC, Strzalka-Mrozik B, Kimsa MW, Gola J, Kochanska-Dziurowicz A, Zebrowska A, et al Differential expression of inflammation-related genes after intense exercise. Prague medical report. 2014;115(1–2):24–32. 10.14712/23362936.2014.3 24874932

[6] Radom-Aizik S, Zaldivar F Jr., Leu SY, Galassetti P, Cooper DM. Effects of 30 min of aerobic exercise on gene expression in human neutrophils. J Appl Physiol (1985). 2008;104(1):236–43. 18006867 10.1152/japplphysiol.00872.2007

[7] Maltseva DV, Ryabenko EA, Sizova SV, Yashin DV, Khaustova SA, Shkurnikov MY. Effect of exercise on the expression of HSPBP1, PGLYRP1, and HSPA1A genes in human leukocytes. Bulletin of experimental biology and medicine. 2012;153(6):866–8. 23113305 10.1007/s10517-012-1846-x

[8] Żychowska M, Kemerytė-Riaubienė E, Gocentas A, Jascaniniene N, Chruściński G. Changes in leukocyte HSPA1A, HSPB1 mRNA in basketball players after plyometric training. Balt J Health Phys Act. 2016;8 (1):18–24.

[9] Morimoto RI, Tissieres A, Georgopoulos C. The biology of heat shock proteins and molecular chaperones: Cold Spring Harbor Laboratory Press; 1994.

[10] Welch WJ. Mammalian stress response: cell physiology, structure/function of stress proteins, and implications for medicine and disease. Physiological reviews. 1992;72(4):1063–81. 1438579 10.1152/physrev.1992.72.4.1063

[11] Buttner P, Mosig S, Lechtermann A, Funke H, Mooren FC. Exercise affects the gene expression profiles of human white blood cells. J Appl Physiol (1985). 2007;102(1):26–36. Epub 2006/09/23. 16990507 10.1152/japplphysiol.00066.2006

[12] Donnikov AE, Shkurnikov MU, Akimov EB, Tonevitsky AG. Relationship between the degree of cardiovascular adaptation and Th1/Th2 polarization of immune response. Bulletin of experimental biology and medicine. 2008;146(4):462–5. 19489321 10.1007/s10517-009-0334-4

[13] Ryan AJ, Gisolfi CV, Moseley PL. Synthesis of 70K stress protein by human leukocytes: effect of exercise in the heat. J Appl Physiol (1985). 1991;70(1):466–71. 2010406 10.1152/jappl.1991.70.1.466

[14] Sakharov DA, Maltseva DV, Riabenko EA, Shkurnikov MU, Northoff H, Tonevitsky AG, et al Passing the anaerobic threshold is associated with substantial changes in the gene expression profile in white blood cells. European journal of applied physiology. 2012;112(3):963–72. Epub 2011/07/01. 10.1007/s00421-011-2048-3 21717121

[15] Jastrzebski Z, Zychowska M. Effects of 6-week specific low-intensity training on selected aerobic capacity parameters and HSPA1A, HSPB1, and LDHb gene expression in high-level rowers. Genetics and molecular research: GMR. 2015;14(3):7538–47. Epub 2015/07/28. 10.4238/2015.July.3.29 26214432

[16] Ecochard L, Lhenry F, Sempore B, Favier R. Skeletal muscle HSP72 level during endurance training: influence of peripheral arterial insufficiency. Pflugers Archiv: European journal of physiology. 2000;440(6):918–24. 11041559 10.1007/s004240000362

[17] Jastrzębski Z, Żychowska M. Are changes in HSPA1A, HSPB1 and LDH-b genes expression during physical performance „till exhaustion” independent of their exercise possibility? Baltic Journal of Health & Physical Activity. 2014;6(4):252–8.

[18] Fehrenbach E, Passek F, Niess AM, Pohla H, Weinstock C, Dickhuth HH, et al HSP expression in human leukocytes is modulated by endurance exercise. Med Sci Sports Exerc. 2000;32(3):592–600. 10731000 10.1097/00005768-200003000-00007

[19] Zeibig J, Karlic H, Lohninger A, Damsgaard R, Smekal G. Do blood cells mimic gene expression profile alterations known to occur in muscular adaptation to endurance training? Eur J Appl Physiol. 2005;95(1):96–104. 10.1007/s00421-005-1334-3 15815935

[20] Ziemann E, Zembron-Lacny A, Kasperska A, Antosiewicz J, Grzywacz T, Garsztka T, et al Exercise training-induced changes in inflammatory mediators and heat shock proteins in young tennis players. Journal of sports science & medicine. 2013;12(2):282–9. 24149807 PMC3761842

[21] Leggate M, Nowell MA, Jones SA, Nimmo MA. The response of interleukin-6 and soluble interleukin-6 receptor isoforms following intermittent high intensity and continuous moderate intensity cycling. Cell stress & chaperones. 2010;15(6):827–33. Epub 2010/04/17. 20396982 10.1007/s12192-010-0192-z PMC3024071

[22] Lira FS, Panissa VL, Julio UF, Franchini E. Differences in metabolic and inflammatory responses in lower and upper body high-intensity intermittent exercise. Eur J Appl Physiol. 2015;115(7):1467–74. Epub 2015/02/18. 10.1007/s00421-015-3127-7 25688040

[23] Dallas G, Zacharogiannis E, Paradisis G. Physiological profile of elite Greek gymnasts. Journal of Physical Education and Sport. 2013;13(1):27–32.

[24] Bencke J, Damsgaard R, Saekmose A, Jorgensen P, Jorgensen K, Klausen K. Anaerobic power and muscle strength characteristics of 11 years old elite and non-elite boys and girls from gymnastics, team handball, tennis and swimming. Scandinavian journal of medicine & science in sports. 2002;12(3):171–8. 12135450 10.1034/j.1600-0838.2002.01128.x

[25] Goswami A, Gupta S. Cardiovascular stress and lactate formation during gymnastic routines. The Journal of sports medicine and physical fitness. 1998;38(4):317–22. 9973775

[26] Jemmi M, Friemel F, Lechevalier J-M, Origas M. Heart rate and blood lactate concetration analysis during a high-level men’s gymnastics competition. J Strength Cond Assoc. 2000;14(4):389–94.

[27] Bassa H, Kotzamanidis C, Siatras T, Mameletzi DS, D. Coactivation of knee muscles during isokinetic concentric and eccentric knee extensions and flexions in prepubertal gymnasts. Isokinet Exerc Sci. 2002;10(3):137–44.

[28] Bar-Or O. TheWingate anaerobic test: an update on methodology, reliability and validity. Sports Medicine. 1987;4(6):381–94. 3324256 10.2165/00007256-198704060-00001

[29] Dotan R, Bar-Or O. Load optimization for the Wingate Anaerobic Test. Eur J Appl Physiol O. 1983;51(3):409–17. 10.1007/BF00429077 6685039

[30] Chomczynski P, Sacchi N. Single-step method of RNA isolation by acid guanidinium thiocyanate-phenol-chloroform extraction. Analytical biochemistry. 1987;162(1):156–9. 10.1006/abio.1987.9999 2440339

[31] Schmittgen TD, Livak KJ. Analyzing real-time PCR data by the comparative C(T) method. Nature protocols. 2008;3(6):1101–8. 18546601 10.1038/nprot.2008.73

[32] Jemni M, Sands WA, Friemel F, Stone MH, Cooke CB. Any effect of gymnastics training on upper-body and lower-body aerobic and power components in national and international male gymnasts? J Strength Cond Res. 2006;20(4):899–907. 10.1519/R-18525.1 17149990

[33] Franchini E, Takito MY, Kiss MAPDM, S. S. Physical fitness and anthropometrical differences between elite and non-elite judo players. Biol Sport. 2005;22(4):315–28.

[34] Gierczuk D, Hübner-Woźniak E, Długołęcka B. Influence of training on anaerobic power and capacity of upper and lower limbs in young greco-roman wrestlers. Biol Sport. 2012;29(3):235–9.

[35] Morton JP, Kayani AC, McArdle A, Drust B. The exercise-induced stress response of skeletal muscle, with specific emphasis on humans. Sports Med. 2009;39(8):643–62. Epub 2009/09/23. 10.2165/00007256-200939080-00003 19769414

[36] Neubauer O, Sabapathy S, Lazarus R, Jowett JB, Desbrow B, Peake JM, et al Transcriptome analysis of neutrophils after endurance exercise reveals novel signaling mechanisms in the immune response to physiological stress. J Appl Physiol (1985). 2013;114(12):1677–88. 23580600 10.1152/japplphysiol.00143.2013

[37] Farzad B, Gharakhanlou R, Agha-Alinejad H, Curby DG, Bayati M, Bahraminejad M, et al Physiological and performance changes from the addition of a sprint interval program to wrestling training. Journal of strength and conditioning research / National Strength & Conditioning Association. 2011;25(9):2392–9. 10.1519/JSC.0b013e3181fb4a33 21849912

[38] Ravier G, Dugue B, Grappe F, Rouillon JD. Impressive anaerobic adaptations in elite karate athletes due to few intensive intermittent sessions added to regular karate training. Scandinavian journal of medicine & science in sports. 2009;19(5):687–94. Epub 2008/08/13. 18694436 10.1111/j.1600-0838.2008.00807.x

[39] Franchini E, Julio UF, Panissa VL, Lira FS, Gerosa-Neto J, Branco BH. High-Intensity Intermittent Training Positively Affects Aerobic and Anaerobic Performance in Judo Athletes Independently of Exercise Mode. Frontiers in physiology. 2016;7:268 Epub 2016/07/23. 10.3389/fphys.2016.00268 27445856 PMC4923181

[40] Fuqua JS, Rogol AD. Neuroendocrine alterations in the exercising human: implications for energy homeostasis. Metabolism: clinical and experimental. 2013;62(7):911–21. 23415825 10.1016/j.metabol.2013.01.016

[41] Robbesyn F, Salvayre R, Negre-Salvayre A. Dual role of oxidized LDL on the NF-kappaB signaling pathway. Free radical research. 2004;38(6):541–51. 15346645 10.1080/10715760410001665244

[42] Driessler F, Venstrom K, Sabat R, Asadullah K, Schottelius AJ. Molecular mechanisms of interleukin-10-mediated inhibition of NF-kappaB activity: a role for p50. Clinical and experimental immunology. 2004;135(1):64–73. 10.1111/j.1365-2249.2004.02342.x 14678266 PMC1808913

[43] Hovsepian E, Penas F, Siffo S, Mirkin GA, Goren NB. IL-10 inhibits the NF-kappaB and ERK/MAPK-mediated production of pro-inflammatory mediators by up-regulation of SOCS-3 in Trypanosoma cruzi-infected cardiomyocytes. PloS one. 2013;8(11):e79445 10.1371/journal.pone.0079445 24260222 PMC3832617

[44] Wang P, Wu P, Siegel MI, Egan RW, Billah MM. Interleukin (IL)-10 inhibits nuclear factor kappa B (NF kappa B) activation in human monocytes. IL-10 and IL-4 suppress cytokine synthesis by different mechanisms. The Journal of biological chemistry. 1995;270(16):9558–63. 7721885 10.1074/jbc.270.16.9558

[45] Nieman DC, Henson DA, Davis JM, Dumke CL, Utter AC, Murphy EA, et al Blood leukocyte mRNA expression for IL-10, IL-1Ra, and IL-8, but not IL-6, increases after exercise. Journal of interferon & cytokine research: the official journal of the International Society for Interferon and Cytokine Research. 2006;26(9):668–74. 10.1089/jir.2006.26.668 16978071

[46] Periard JD, Ruell P, Caillaud C, Thompson MW. Plasma Hsp72 (HSPA1A) and Hsp27 (HSPB1) expression under heat stress: influence of exercise intensity. Cell stress & chaperones. 2012;17(3):375–83. 22222935 10.1007/s12192-011-0313-3 PMC3312965

[47] Acunzo J, Katsogiannou M, Rocchi P. Small heat shock proteins HSP27 (HspB1), alphaB-crystallin (HspB5) and HSP22 (HspB8) as regulators of cell death. The international journal of biochemistry & cell biology. 2012;44(10):1622–31. 22521623 10.1016/j.biocel.2012.04.002

[48] Nagaraja GM, Kaur P, Asea A. Role of human and mouse HspB1 in metastasis. Current molecular medicine. 2012;12(9):1142–50. 22804237 10.2174/156652412803306701

[49] Osawa Y, Azuma K, Tabata S, Katsukawa F, Ishida H, Oguma Y, et al Effects of 16-week high-intensity interval training using upper and lower body ergometers on aerobic fitness and morphological changes in healthy men: a preliminary study. Open access journal of sports medicine. 2014;5:257–65. 10.2147/OAJSM.S68932 25395872 PMC4226445

