# Correction: Cellular Stress Response Gene Expression During Upper and Lower Body High Intensity Exercises

**DOI:** 10.1371/journal.pone.0172608

**Published:** 2017-02-15

**Authors:** The PLOS ONE Staff

There are errors in the Funding section. The correct funding information is as follows: This scientific work was funded under the program of Polish Ministry of Science and Higher Education (http://www.nauka.gov.pl/) under the name "Development of Academic Sport" in years 2015–2017 Project No. 0018/RS3/2015/53. The funding organization does not affect the study results.
